# A retrospective analysis of key predictors and patient outcomes: Using artificial intelligence for precision survival prediction in colorectal cancer

**DOI:** 10.3205/000345

**Published:** 2025-08-29

**Authors:** Muayyad M. Ahmad, Eslam Bani Mohammad

**Affiliations:** 1 Clinical Nursing Department, School of Nursing, The University of Jordan, Amman, Jordan; 2 Department of Nursing, Faculty of Nursing, Al-Balqa Applied University, Al-Salt, Jordan

**Keywords:** artificial intelligence, colorectal cancer, survival prediction, mortality, Bayesian network

## Abstract

**Objective:**

The study aims to identify critical predictors of mortality and evaluate the performance of different artificial intelligence (AI) models among patients with colorectal cancer (CRC). Furthermore, the study also sought to enhance our comprehension of survival outcomes by identifying key predictors and evaluating the accuracy of AI-driven prediction methods for patients with CRC.

**Methods:**

The study employed a retrospective-predictive design, using data from the electronic health records of patients with colorectal cancer (CRC) admitted between 2016 and 2023. Among the eight AI models created by the SPSS Modeler version 18.0, the Bayesian network model was the most effective of the eight models in this study.

**Results:**

The researchers identified the most relevant variables associated with mortality among patients with CRC through data visualization. The study analysed 1,159 colorectal cancer patients, with 45.7% living up to six years and 54.3% living between seven and 16 years post-diagnosis. The Bayesian network AI model identified stage, age, recurrence, sex, marital status, and smoking status as key predictors.

**Conclusion:**

This study model’s structure emphasizes these predictors’ interconnectedness because parent nodes directly connect to child nodes. The model shows how age, smoking status, marital status, cancer stage, and recurrence affect patient survival. The model clarifies these variables’ interactions.

## Introduction

Colorectal cancer (CRC) is one of the leading causes of cancer deaths globally, and recurrence risk affects patient survival [1]. According to studies, recurrent CRC patients’ median survival duration depends on the location and timing of the CRC recurrence [2], [3]. 

Artificial intelligence (AI) is crucial for health prediction due to its capacity to rapidly and accurately evaluate and process vast quantities of data [4], [5]. Traditional approaches may fail to detect subtle patterns and correlations in complex datasets that AI algorithms can find [6], [7]. Furthermore, traditional health prediction uses statistical techniques that are sometimes limited by human biases and the inability to consider multiple variables [8]. AI excels in managing multidimensional and complex data, resulting in more accurate forecasts and deeper insights. Prediction driven by AI can enhance patient outcomes and save lives by enabling early disease detection and treatment [8].

Few studies have shown that AI can improve diagnostic accuracy, treatment planning, and patient care in CRC prediction [9]. For early detection of CRC, Schiele et al. [10] used deep learning algorithms to assess histopathological pictures and predict recurrence. Gonzalez et al. [11] used machine learning algorithms to predict CRC survival and recurrence using Electronic Health Records (EHR) data. Tamang and Kim [12] created an AI-based approach, which has improved polyp identification rates over traditional approaches, minimizing misses and enhancing early diagnosis. 

The survival rates of CRC for men and women are significantly different [13]. According to research, women have better CRC survival rates than men [14]. Due to genetic differences in tumor biology and hormonal variables, females often have less severe cancer types [14], [15]. Behavioral and lifestyle factors, including screening and treatment adherence, can affect survival results [16]. Other research shows that while women have better survival rates, they often have more advanced CRC upon diagnosis than men [13]. Male patients are diagnosed earlier and may proceed more aggressively [14]. 

Age, which reflects the patient’s health and disease stage upon diagnosis, affects CRC survival. Younger CRC patients have a more aggressive disease and a worse prognosis, according to research [17]. Despite being discovered later due to lower screening rates and comorbidities, elder people usually have a less aggressive disease [18], [19]. However, due to cancer stage and therapy access, senior people may have better survival rates [20]. 

According to research, married people have better five-year survival rates than single people [21]. This outcome is possibly due to better support systems, treatment adherence, and psychological well-being [22]. Social support from a spouse or partner can help a patient manage the disease, follow treatment, and preventive measures, improving survival [23]. 

Non-smokers have a decreased chance of CRC, less severe illness, and better survival [24]. Recent Cancer Research findings link smoking to a higher rate of colon cancer and more aggressive tumor behavior, which lowers survival rates [25]. The carcinogenic effects of tobacco, which accelerate tumor progression and metastasis, raise the chance of smokers developing advanced-stage disease and having greater treatment response problems [26], [27]. Smoking can also reduce cancer treatment efficacy and exacerbate treatment-related problems, lowering survival rates [26]. 

Earlier TNM (Tumor, Node, and Metastasis) stages at diagnosis greatly improve CRC survival [28]. Gastroenterology studies show that patients with stage I and stage II CRC have a higher five-year survival rate [29]. Due to early treatment and lower illness severity at diagnosis, these patients often have a better quality of life and longer survival rate [29]. 

## Objectives

This study aimed to examine the AI models in predicting the duration of life from the time of CRC diagnosis until mortality. The study also sought to enhance our comprehension of survival outcomes by identifying key predictors and evaluating the accuracy of AI-driven prediction methods for patients with CRC.

## Methods

### Study design

The study employed a retrospective-predictive and design, using data from the EHR of patients admitted between 2016 and 2023.

### Eligibility criteria

The necessary variables were implemented to retrieve the records of patients with CRC from the chosen institution. The inclusion criteria consist of data regarding patients who died with CRC. 

### Data processing

The data acquisition procedure was completed in four months after consent was obtained to extract the necessary data. To identify missing values, outliers, and inconsistencies, the data were subjected to frequency analysis and inspection. The retrieved data was sorted, cleaned, and structured, resulting in the elimination of redundant material. The Statistical Package for Social Sciences, version 29.1, was employed to conduct descriptive statistics [30]. 

The researchers identified the most relevant variables associated with mortality among patients with CRC through data visualization. Furthermore, SPSS Modeler version 18.0 was employed to visualize, analyze, and administer the data [31]. This software has the capacity to manage data for descriptive and predictive modeling, as well as to display data with high statistical power. 

As shown in Table 1 (Tab. 1), eight AI models were generated to predict the length of time lived after diagnosis with CRC in relation to the study variables. Based on the area under the curve (AUC) (0.845) and the overall accuracy of 81.828%, the Bayesian network model was selected to examine the predictors and the probabilities for age lived until death among patients with CRC.

### Ethical consideration

The study was authorized by the Scientific Research Committees at The University of Jordan. Additionally, the study facility’s ethical committee granted permission for data acquisition under the reference number (IRB-JUH 10/24/1503; dated on 16/1/2024). Patient information was managed with assurance by employing an ID as a unique identifier for each record. 

## Results

A total of 1,159 patients with CRC who died within one to 16 years of their diagnosis comprised the study sample. The duration of the study sample’s life after being diagnosed with CRC was divided into two groups: one group, which lived up to six years (n=530, 45.7%), and the other group, which lived between seven and 16 years (n=629, 54.3%). The study sample consisted of slightly more males (55.9%, n=648). The largest cohort consisted of patients aged 65 to 79 years (37.3%). The number of nonsmokers (n=674, 58.2%) was greater than that of smokers. The sample consisted of approximately 83% married individuals. Most of the patients (62.3%) were in stage 4, while 26% were in stage 3, as per the TNM staging system. The disease recurred in only 25% of the patients following treatment (Table 2 (Tab. 2)).

**Figure 1:**
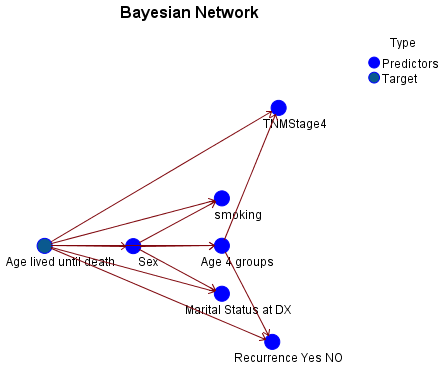
The Bayesian model with the study variables’ interrelationship

The Bayesian network model (Figure 1 (Fig. 1)) illustrates the relationships between the outcome variable (length of time lived before death, 1–6 years versus 7–16 years) and the six predictors. The graphics-based Bayesian network model shows probabilistic links between variables. A Bayesian network parent node has direct edges to one or more child nodes. The child node’s status depends conditionally on its parents. Predecessor nodes affect offspring probability distribution. The link between parent-child pairs, caused by the parent nodes’ different conditions, suggests the child node’s probability evolution [32].

**Figure 2:**
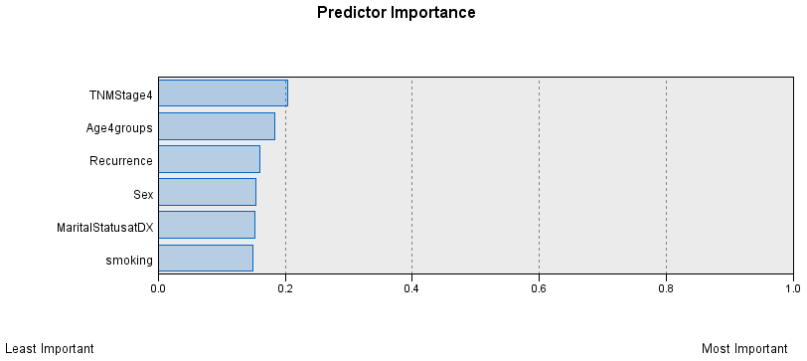
The study predictors’ importance in the Bayesian model

The AI model identified six predictors regarding the studied outcome (length of time lived before death), as illustrated in Figure 2 (Fig. 2). The TNM stages are the most significant predictor, followed by the age categories, recurrence of CRC, sex of individuals, marital status, and smoking status. 

The Bayesian network models with predictor variables and the probabilities of the length of time lived before death are illustrated in Table 3 (Tab. 3). The conditional probability was 0.46 for individuals who lived for 1 to 6 years and 0.54 for those who lived for 7 to 16 years. The conditional probability of death within 1 to 6 years was 83% for females who did not smoke, compared to 42% for males. Regarding the duration of time until mortality, males had conditional probabilities of 0.93 and 0.92, respectively, while married females had identical probabilities of 0.72. The highest conditional probabilities that resulted from the parent nodes’ sex of patients and the length of time before death were 0.42 for males aged 65 to 79 and 0.38 for females in the same age group. In general, the probability of males living for 7 to 16 years was higher than that of females (0.58). The age group with the earliest age group had a 0.75 probability of living 1 to 6 years and was in TNM stage 4. The age group 65–79 and TNM stage 4 had a 0.71 probability of living 1 to 6 years. The youngest age group, which lived from 1 to 6 years and 7 to 16 years, had the highest conditional probabilities of recurrence (0.31 and 0.32).

## Discussion

The study developed eight AI models to predict the duration of life following a CRC diagnosis and represents a substantial advancement in the application of machine learning to oncology. The Bayesian network model, which was chosen for its overall accuracy of 81.828% and area under the curve (AUC) of 0.845, demonstrates a robust predictive quality. The incorporation of a diverse array of predictors, such as age, smoking status, marital status, cancer stage, and recurrence, facilitates a broad assessment of factors that affect survival outcomes. 

The conditional probabilities of survival for patients with CRC are comprehensively understood by the Bayesian network model introduced in this study. Similar to a study conducted by Ghebrial et al. [33], our model illustrates that there are significant disparities in survival probabilities, with nonsmoking females having a substantially higher likelihood of mortality within 1 to 6 years than their male counterparts. Additionally, the model suggests that males have a higher probability of surviving for 7 to 16 years, particularly in the 65 to 79 age group. These findings emphasize the complexity of predicting patient outcomes, offering an understanding of the interaction between demographic and clinical factors in the context of CRC survival [34].

Traditional studies frequently emphasize the significant impact of early detection and treatment advancements on the enhancement of CRC survival rates, particularly for stage 3 and 4 patients [35], [36], [37]. However, the high conditional probabilities of mortality within 1 to 6 years for both younger and older patients in stage 4 in this study suggest that late-stage CRC continues to present significant survival challenges, despite the progress that has been made. Furthermore, and consistent with a study by Gheybi et al. [38], the finding that the risk of recurrence is highest in the youngest age cohort within both survival brackets (1–6 years and 7–16 years) may indicate that the model fails to adequately account for the underlying biological factors or disparities in treatment efficacy. This emphasizes the importance of conducting further research to investigate the potential benefits of personalized treatment strategies and age-specific recurrence mechanisms.

The study’s findings indicate that nearly half of the patients diagnosed with CRC survived for a period of up to six years, while a slightly larger proportion survived for a period of seven to sixteen years. This division implies that the survival outcomes of CRC patients are subject to significant variation, which may be influenced by a variety of factors, such as the stage of the disease at diagnosis, the efficacy of treatment, genetic predispositions, and overall health [15]. The predominance of males and the largest cohort in the age group of 65 to 79 years emphasize the demographic trends that are consistent with the existing literature, which recognizes the significant role of gender and older age in the prognosis of CRC [3], [33]. 

However, there are certain arguments that can be made when comparing these results to the broader literature. This study does not provide information regarding the distribution of cancer stages at the time of diagnosis or the specific treatments that were implemented, even though numerous studies have emphasized the importance of early detection and sophisticated treatment options in improving survival rates [39]. Furthermore, the results of the study suggest that the sample population is predominantly composed of married individuals and nonsmokers, with most of the stage 4 CRC cases. The increased proportion of nonsmokers is in accordance with specific literature that implies that nonsmokers may experience superior health outcomes and longevity because of the absence of smoking-related complications following a diagnosis [24]. In contrast to other studies, which frequently report higher recurrence rates for late-stage CRC [1], the recurrence rate of 25% is relatively modest. This discrepancy may suggest that the efficacy of the treatment, the practices of follow-up, or the characteristics of the sample have varied. 

The Bayesian network model’s graphical representation and accuracy are compelling. To ensure that the predictions are consistent with real-world outcomes and can be trusted in clinical practice, they should be validated against broader datasets and integrated with clinical expertise. Furthermore, the Bayesian network model demonstrates potential; consequently, it is essential to execute a critical evaluation of its outcomes in comparison to the current body of literature. Traditional models frequently underscore the importance of early detection and the role of specific treatment modalities in improving survival rates. To ensure that the models are not only statistically valid but also effectively applicable in a diverse array of healthcare environments, the existing literature frequently suggests the integration of clinical expertise with AI predictions. The implementation of AI models, such as the Bayesian network, should be meticulously evaluated and compared to the broader clinical experiences and outcomes documented in the literature, even though they provide substantial advancements. 

Even though the Bayesian network model offers valuable probabilistic insights, it is imperative that its predictions are tested through comprehensive longitudinal studies and interpreted in conjunction with clinical expertise to ensure that they are consistent with real-world patient outcomes and effectively contribute to clinical decision-making. 

## Conclusion

The Bayesian network model employed in this study illustrates the probabilistic relationships between the duration of time lived after a CRC diagnosis and six critical predictors. The model visually represents the relationships between various variables, such as age, smoking status, marital status, cancer stage, and recurrence, to influence patient survival. This is achieved using graphics-based nodes and edges. The model provides a clear comprehension of the interactions between these variables. The model’s structure emphasizes the interconnectedness of these predictors, as parent nodes have direct interconnections to child nodes and the status of the child node is conditionally dependent on its parents’ node. By facilitating analysis of how changes in one variable can probabilistically alter the outcomes in another, this method facilitates a more comprehensive and dynamic comprehension of survival probabilities.

## Notes

### Authors’ ORCIDs

- Muayyad M. Ahmad: 0000-0002-4388-8332
- Eslam Bani Mohammad: 0000-0003-3569-7875

### Competing interests

The authors declare that they have no competing interests.

## Figures and Tables

**Table 1:**
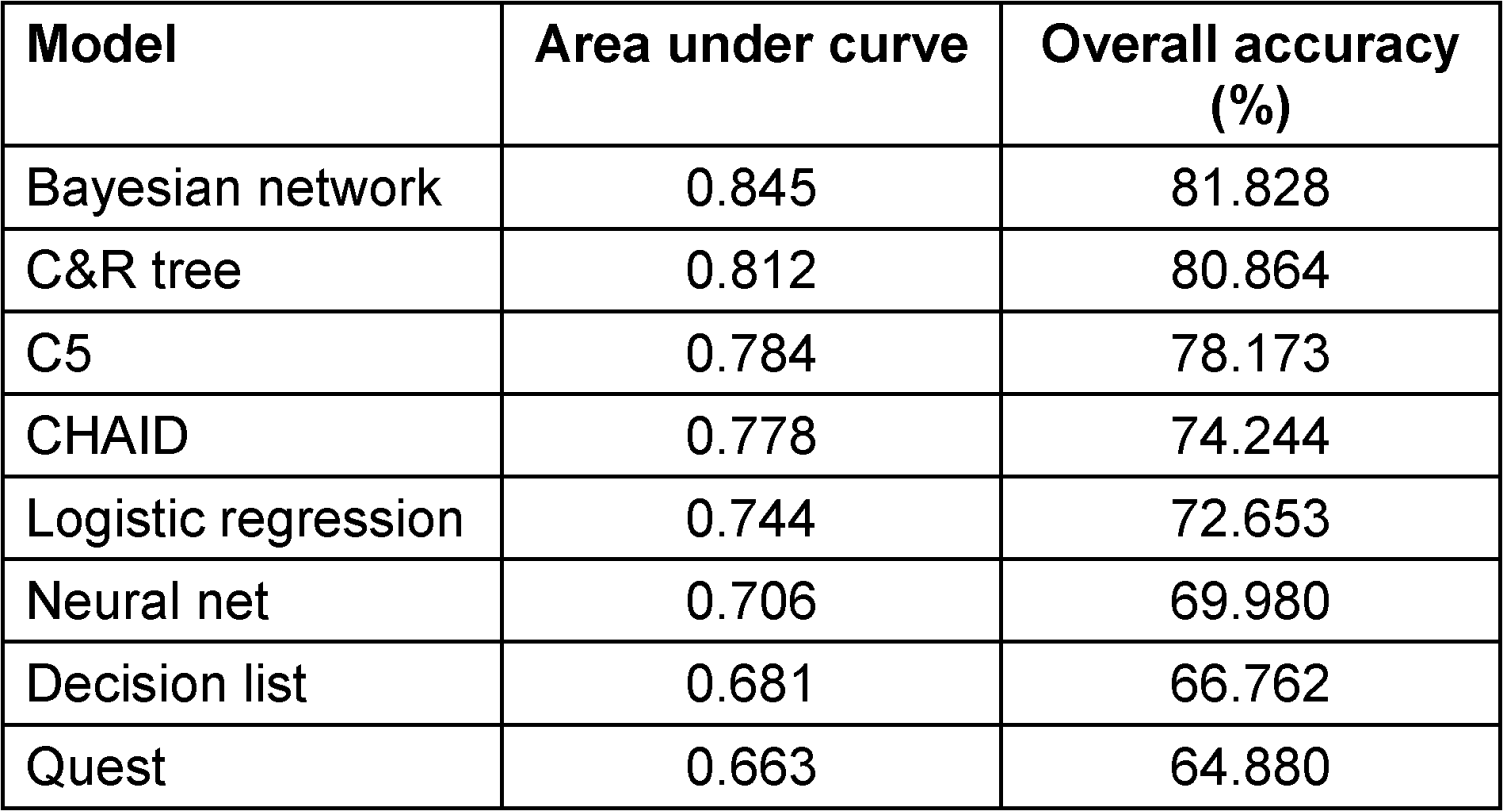
The eight models created from the study data

**Table 2:**
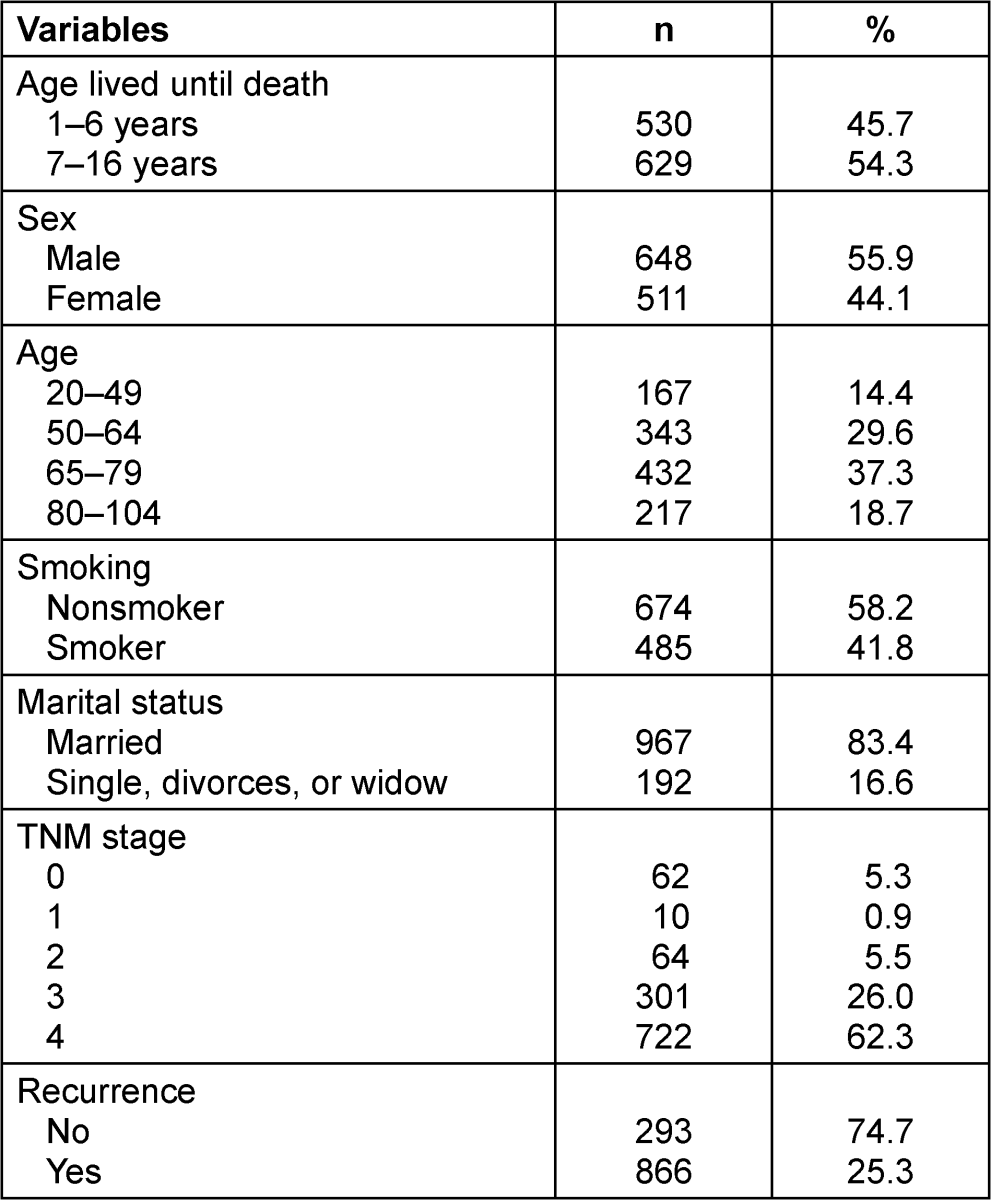
Descriptive statistics for the study variables (N=1,159)

**Table 3:**
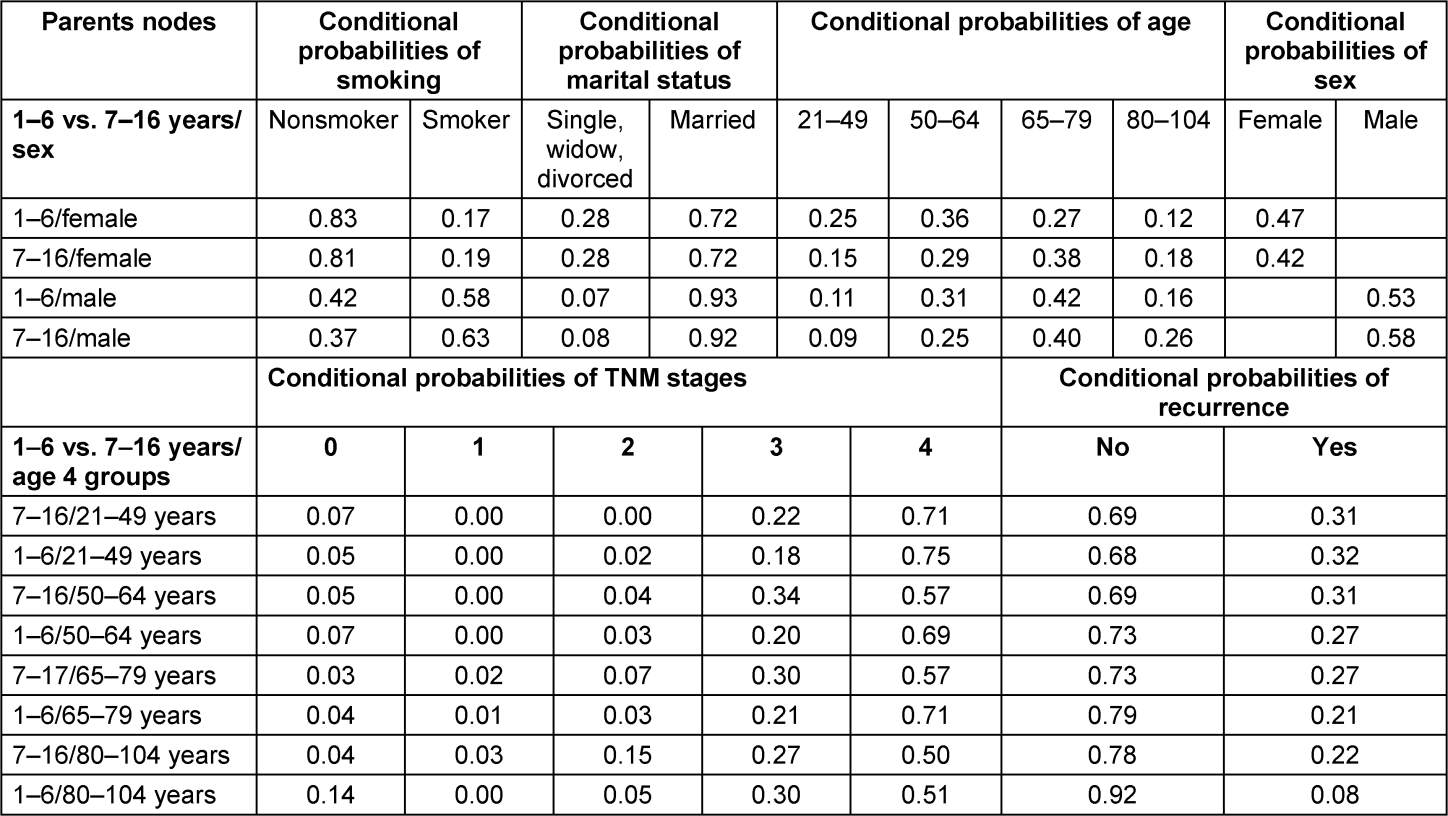
The Bayesian network model’s predictors and the probabilities for age lived until death among patients with CRC
